# The biological functions of DNA: from the sublime to the slime

**DOI:** 10.1186/s13075-017-1464-0

**Published:** 2017-12-15

**Authors:** David S. Pisetsky

**Affiliations:** Medicine and Immunology, Duke University Medical Center, Medical Research Service, Durham VA Medical Center, Durham, North Carolina 27710 USA

**Keywords:** DNA, Biofilms, Bacteria, Anti-DNA, Lupus, Toll-like receptor, Cell death, Amyloid

Of all the molecules in nature, DNA is the most exalted. The blueprint of life, DNA bears the genetic code inscribed in the famous double helix. The structure of DNA is elegant, magisterial, even sublime. In the conceptualization of DNA’s role in biology, two overarching ideas have long dominated. First, DNA is an informational macromolecule. Second, DNA’s locus of action is intracellular. In the past decades, this conceptualization has undergone a paradigm shift that has left many investigators positively shaking.

The first evidence for a function of DNA beyond genetics came with the recognition that bacterial DNA containing CpG motifs can act as a PAMP (pathogen associated molecular pattern) to stimulate innate immunity. While CpG motifs (an unmethylated cytokine guanosine dinucleotide) are particularly active, a requirement for a CpG motif can be overcome by the binding of DNA to proteins that effectively transfect DNA into cells to interact with both toll-like (TLR) and non-toll-like internal nucleic acid receptors.

Since DNA, like other TLR agonists, can stimulate cells when added to the media of cultured cells, studies on immune stimulation by CpG DNA thus focused attention on the generation of extracellular DNA. As shown in both in vitro and in vivo systems, DNA can translocate from the inside to the outside of the cell during cell death in processes that include apoptosis, necrosis, and NETosis. Because of these translocation events, levels of extracellular DNA in the blood can rise in conditions such as sepsis, shock, and malignancy.

Extracellular DNA is not just a feature of the mammalian organism, however. Rather, the generation of extracellular DNA occurs prominently during bacterial life and extracellular DNA in soil and water are major constituents of many ecosystems. In these systems, biofilms are a remarkable microenvironment in which bacteria grow as well-integrated communities. In the biofilm, bacteria are surrounded by a matrix or extracellular polymer substance to organize, protect, and nurture the colony and provide a source of nutrients. Biofilms can keep bacteria safe from the outside world and promote resistance to antibiotics and other anti-bacterial attacks.

While the composition of a biofilm may vary by species, in general, the matrix consists of polysaccharides, proteins, and DNA. In organizing the biofilm, DNA plays an important structural role. As a polymer, DNA can confer viscosity and DNA has an extensive capacity for binding of other molecules, both large and small, because of the negative charges on the backbone. Indeed, DNA can make great slime, which is after all what many biofilms are.

The construction of a biofilm matrix depends on a supply of extracellular DNA along with the other constituents. During bacterial growth, the release of DNA can be a highly regulated process involving secretion from cells. Extracellular DNA can also come from cells killed by suicide or by fratricide, with those dying sacrificed for the greater good. DNA from the host (e.g., NETs) may also be incorporated into the biofilm.

This brief account provides important lessons of unexpected facets on DNA’s biology. The first concerns systems to elucidate cell growth and function. Many studies on bacterial physiology have involved culture in liquid media in which so-called planktonic bacteria are sloshed, swirled, and agitated. Not surprisingly, such studies may miss important features that would be apparent for bacteria growing more felicitously and sedately in the safe confines of a biofilm.

A second lesson concerns the commonalities of the eukaryotic and prokaryotic worlds. The release of DNA by dead and dying organisms has striking similarities in the bacterial and mammalian systems. Of interest, in the formation of biofilms, bacterial cell lysis can resemble an explosion and form microparticles from membrane fragments burst from cells. While blebbing may produce vesicles in both mammalian and bacterial systems, the formation of particles may simply reflect events in cell death in which the cell body essentially shatters rather than collapsing or deflating.

A final lesson about biofilms relates to the use of reductionist systems to model complex phenomena. In nature, DNA is probably rarely if ever alone or, in other more suggestive terminology, naked. In certain biofilms, DNA binds to a protein called curli to form amyloids. Interesting, DNA–curli complexes can induce anti-DNA autoantibody responses that have been difficult to elicit by other complexes or adjuvants. Perhaps biofilms are the relevant source of DNA to induce anti-DNA production in lupus, with immunogenicity reflecting the display of DNA in an ensemble of other molecules which can activate more than one TLR simultaneously.

DNA now has three distinct functions—genetics, immunological, and structural—that are widely disparate and variously dependent on the sugar phosphate backbone and the bases. Does it have any others? Who knows? I say let’s wait and see if this extraordinary molecule has any more hidden identities to reveal.
